# Awareness, Knowledge, and Perceptions of Neglected Tropical Diseases among Personnel of a Tertiary Hospital in Makurdi, Nigeria: Controlling Neglected Tropical Diseases

**DOI:** 10.4314/ejhs.v35i5.6

**Published:** 2025-09

**Authors:** Nndunno Asheku Akwaras, David Aondona Daniel, Tongriang Ben-Ameh, Laadi Terrumun Swende, Bamidele Ohiozoje Ornguga, Grace Nwunuji Rimamnunra

**Affiliations:** 1 Department of Family Medicine, Federal Medical Centre, Makurdi, Benue State, Nigeria; 2 Department of Ophthalmology, College of Health Sciences, Benue State University Makurdi, Benue State, Nigeria; 3 Department of Epidemiology and Community Health, College of Health Sciences, Benue State University Makurdi, Benue State, Nigeria

**Keywords:** Awareness, communicable diseases, disease control, knowledge, Neglected Tropical Diseases

## Abstract

**Background:**

Neglected Tropical Diseases (NTDs) are a group of 20 diverse communicable diseases of significant public health concern. Adequately trained personnel play a vital role in implementing effective NTD control programs, as their expertise directly influences patient outcomes and the overall success of intervention strategies. This study assessed the awareness, knowledge, and perceptions of NTDs among staff in a tertiary healthcare facility in Benue State, Nigeria.

**Methods:**

A cross-sectional descriptive design was employed to study 310 randomly selected respondents using an interviewer-administered, semi-structured questionnaire. Data collected were analyzed using descriptive and inferential statistical techniques.

**Results:**

The majority of respondents were aged 31–50 years (75.4%), predominantly female (54.8%), married (72.9%), and had tertiary education (97.4%). Most were health-related professionals (59.7%). Overall, 72.6% of respondents were aware of NTDs, but only 42.6% demonstrated good knowledge. The main source of information was schools/seminars (85.8%). However, only 18.5% could name at least one NTD. Health-related disciplines significantly influenced knowledge, including recognition of NTDs as public health issues (P = 0.030) and awareness of vaccine-preventable NTDs (P = 0.001). Preventive measures such as health education and improved sanitation were widely endorsed.

**Conclusion:**

While awareness of NTDs was relatively high, good knowledge remained suboptimal, particularly among non-health-related professionals. Health-related disciplines significantly influenced both awareness and knowledge, underscoring the need for targeted educational interventions in non-health sectors. Strengthening public health education, integrating NTD-related content into broader curricula, and prioritizing funding for NTD control measures are recommended to bridge knowledge gaps and promote effective prevention strategies.

## Introduction

Neglected Tropical Diseases (NTDs) constitute a critical global public health concern, disproportionately affecting populations where poverty, poor sanitation, and limited access to healthcare create favorable conditions for their spread (1). NTDs are a group of 20 diverse communicable diseases identified by the World Health Organization (WHO) as endemic in tropical and subtropical regions. Many of these diseases are vector-borne, have animal reservoirs, and are associated with complex life cycles (1,2). Agricultural practices such as irrigation also contribute to their transmission, particularly schistosomiasis, thereby complicating public health control efforts (3).

These diseases including trachoma, schistosomiasis, lymphatic filariasis, onchocerciasis, and leishmaniasis, affect more than one billion people globally, especially those in underserved and marginalized communities (4-6). Their impact extends beyond health, leading to chronic disability, mental health challenges, social stigma, and economic losses, which perpetuate cycles of poverty and hinder socioeconomic development (4,5).

Despite their burden, NTDs receive relatively limited attention compared with other major public health challenges such as malaria and HIV/AIDS (2). This neglect is compounded by insufficient awareness among healthcare personnel, who play a pivotal role in surveillance, prevention, and management. To address this, WHO developed the NTD Roadmap (2021–2030), aligned with the Sustainable Development Goals (SDGs). The roadmap outlines global targets, including a 90% reduction in people requiring interventions against NTDs, a 75% reduction in NTD-related disability-adjusted life years (DALYs), elimination of at least one NTD in 100 countries, and eradication of dracunculiasis and yaws. It also emphasizes cross-cutting goals such as improved access to water, sanitation, and hygiene in endemic areas (2,7).

The concept of tool-ready NTDs refers to those with effective, affordable, and safe tools and strategies for control or elimination (6). Such tools include preventive chemotherapy, vector control, and improved sanitation and hygiene. Examples include onchocerciasis (river blindness), schistosomiasis, soil-transmitted helminth infections, and trachoma. This approach has been instrumental in mobilizing global efforts, particularly in resource-limited settings (8). Although many NTDs lack effective vaccines, some are vaccine-preventable (e.g., dengue fever and rabies), while others such as hookworm infection, schistosomiasis, and leishmaniasis currently have vaccines in development (9).

Comprehensive knowledge of NTDs is essential in tertiary healthcare facilities, which serve as referral centers and hubs for specialized care. The awareness of both clinical and nonclinical staff in these facilities influences the effectiveness of NTD control programs, patient outcomes, and broader public health strategies.

In Nigeria, significant gaps in knowledge and awareness of NTDs have been documented among healthcare workers and the general public, though data specific to Benue State remain scarce. For example, a study in Kaduna found that only 57.2% of primary healthcare workers had good knowledge of NTDs (10). Similarly, in Abuja, only 63.1% of respondents demonstrated good knowledge of NTDs and related control activities (11). These findings highlight the urgent need for enhanced education, training, and resource allocation to strengthen awareness and management capabilities among healthcare workers and communities (12).

Poor awareness among hospital personnel is particularly concerning in Nigeria, where the healthcare system is already overburdened by both communicable and non-communicable diseases. Against this backdrop, this study sought to assess awareness of NTDs among personnel in a tertiary healthcare facility in Benue State, Nigeria. Findings from this study are expected to support effective NTD control and elimination strategies while strengthening the capacity of the healthcare system to serve vulnerable populations.

## Materials and Methods

Study site: The study was conducted in Makurdi, the capital of Benue State, North-central Nigeria. Makurdi is an urban settlement. Participants were drawn from staff of the Federal Medical Centre, Makurdi, a 400-bed tertiary hospital. The study population included both clinical staff (doctors, pharmacists, nutritionists, physiotherapists, and medical record staff) and non-clinical staff (administrative officers, accountants, cleaners, drivers, and maintenance personnel).

**Study design**: A cross-sectional study design was employed. Leslie-Kish formula for a single proportion was used to calculate the minimum sample size. The calculation yielded a sample size of 302. To account for possible incomplete responses, an additional 10% (30) was added, resulting in a final sample size of 332. Of these, 310 participants completed the study.

**Inclusion and exclusion criteria**: All consenting staff were included, while those actively attending to emergencies were excluded.

**Study tool**: Data were collected using a self-administered, pretested questionnaire developed by the researchers after a literature review (8–15). The questionnaire captured socio-demographic characteristics, knowledge of NTDs, and prevention practices. Ten questions (yes/no, “I don't know,” and short-answer) were used to assess NTD knowledge (questions 1, 2, 6–13 in Table 2). Each correct response scored one point; incorrect or “I don't know” responses scored zero. Scores ranged from 0–10, with ≥6 considered good knowledge and <6 considered poor knowledge.

**Data analysis**: Data were analyzed using IBM SPSS version 23. Qualitative variables were summarized with frequencies and percentages, while quantitative variables were summarized with means and standard deviations. Comparisons between groups were made using chi-square tests. A p-value ≤ 0.05 was considered statistically significant.

**Ethical considerations**: The study adhered to institutional ethical standards of the Federal Medical Centre, Makurdi, and the Helsinki Declaration of 1975, revised in 2000. Informed consent was obtained from all participants, and confidentiality was maintained. Ethical approval was granted by the Health Research Ethics Committee of the Federal Medical Centre, Makurdi, Benue State.

## Results

**Sociodemographic characteristics of respondents**: The majority of respondents were aged 31–40 years (37.7%, n=117) and 41–50 years (37.7%, n=117), while only 6.5% were ≤30 years. Females constituted 54.8% (n=170). Most participants were married (n=226, 72.9%), while 24.8% (n=77) were single. Respondents were predominantly of Tiv ethnicity (n=111, 35.8%), and all (100.0%) were Christians, with no representation from Islam or other religions.

Nearly all respondents (97.4%, n=302) had tertiary education. More than half (59.7%, n=185) were from health-related disciplines, while 40.3% (n=125) were in non-health-related fields. Regarding income, 54.8% (n=170) earned above 200,000 naira, whereas 12.9% (n=40) reported monthly incomes between 50,000–99,999 naira. (Table 1).

**Table 1:** Sociodemographic characteristics of the respondents

Variable	Frequency (n=310)	Percent
**Age**		
≤ 30	20	6.5
31 – 40	117	37.7
41 – 50	117	37.7
≥ 51	56	18.1
**Gender**		
Male	140	45.2
Female	170	54.8
**Marital status**		
Single	77	24.8
Married	226	72.9
Others	7	2.3
**Ethnicity**		
Tiv	111	35.8
Idoma	109	35.2
Others	90	29.0
**Educational level**		
Tertiary	302	97.4
Secondary	8	2.6
**Type of discipline**		
Health-related	185	59.7
Not health related	125	40.3
**Income level**		
50,000 - 99,999	40	12.9
100,000 - 199,000	100	32.3
> 200,000	170	54.8

**Table 2:** Responses to questions on knowledge about NTDs

Variable	Frequency	Percent
Aware of NTD		
Yes	225	72.6
No	85	27.4
Knows the meaning of acronym NTD (n=225)		
Correct	165	73.3
Incorrect	60	26.6
Source of information on NTD (n=225)		
School/seminars	193	85.8
News	18	8.0
Social media	14	6.2
Has seen someone with NTD? (n=225)		
Yes	137	60.9
No	88	39.1
Are all NTDs public health problems (n=225)		
Yes	141	62.7
No	84	37.3
Knows any NTD programme (n=225)		
Yes	151	67.1
No	74	32.9
Name three NTD control programmes (n=151)		
Named one	28	18.5
Named two	37	24.5
Named three/more	29	19.2
Incorrect names	57	37.8
How many NTDs are there? (n=225)		
17	33	14.7
20 (correct answer)	12	5.3
I don't know	180	80.0
How many NTDs are considered ‘tool ready’? (n=225)		
7 (correct answer)	18	8.0
9	112	49.8
I don't know	95	42.2
Do you know any ‘tool ready’ NTD? (n=225)		
Yes	47	20.9
No	178	79.1
If yes to above, name five tool ready NTDs (n=47)		
Named at least one	27	57.4
Named at least two	10	21.3
Named at least three and above	10	21.3
How many of the NTDs is/are vaccine preventable? (n=225)		
1	14	6.2
3 (correct answer)	4	1.8
I don't know	207	92.0
Aware of the ‘overarching 2030 global targets’ concerning NTDs (n=225)		
Yes	26	11.6
No	199	88.4

Responses to knowledge questions about NTDs: Out of all respondents, 72.6% (n=225) reported being aware of NTDs, while 27.4% (n=85) were not. Of those aware, 73.3% (n=165) correctly identified the meaning of the acronym NTD, while 26.6% (n=60) did not.

The majority (85.8%, n=193) cited schools or seminars as their main source of information. Fewer reported news (8.0%, n=18) or social media (6.2%, n=14). Over half (60.9%, n=137) had seen someone suffering from an NTD, while 39.1% (n=88) had not.

A total of 62.7% (n=141) believed all NTDs are public health problems, while 37.3% (n=84) disagreed. Awareness of NTD control programmes was reported by 67.1% (n=151); however, when asked to name them, only 18.5% (n=28) named one, 24.5% (n=37) named two, 19.2% (n=29) named three or more, and 37.8% (n=57) gave incorrect answers.

Knowledge of technical aspects was low: 80.0% (n=180) were unsure of the total number of NTDs, and only 8.0% (n=18) correctly identified the number of tool-ready NTDs. Just 20.9% (n=47) could name at least one tool-ready NTD, while 79.1% (n=178) could not. Of those who named tool-ready NTDs, 57.4% (n=27) listed one, 21.3% (n=10) listed two, and 21.3% (n=10) listed three or more.

Knowledge about vaccine-preventable NTDs was very poor: 92.0% (n=207) did not know the number, and only 1.8% (n=4) answered correctly. Similarly, only 11.6% (n=26) were aware of the global NTD targets for 2030, while 88.4% (n=199) were not.

**Knowledge of NTDs among respondents**: Among those aware of NTDs, 42.6% demonstrated good knowledge, while 57.3% had poor knowledge. Mean knowledge scores were significantly higher among those with good knowledge (6.4 ± 2.5) compared to those with poor knowledge (3.9 ± 1.5; p = 0.018).

**Responses on prevention and control of NTDs**: Respondents strongly supported systemic interventions: equitable healthcare, health education, poverty reduction, improved sanitation, and increased hospital funding—all receiving between 68.7% and 75.2% “Yes” responses.

In contrast, only 53.2% endorsed “Legislation on NTD prevention.” For inappropriate strategies, fewer respondents agreed: 16.1% for “Change of government” and 24.2% for “Curtailing people's movement.

**Association between discipline and knowledge**: As shown in Table 4, significant associations were observed between discipline type and several knowledge variables, including recognizing NTDs as public health problems (p = 0.030), knowledge of control programmes (p = 0.004), total number of NTDs (p = 0.005), tool-ready NTDs (p = 0.043), and vaccine-preventable NTDs (p = 0.001). Health-related personnel consistently showed greater knowledge and awareness than their non-health-related counterparts.

**Table 4:** Association between type of discipline and knowledge question responses

Variables	Type of discipline		Test statistic	P-value

	Health related n (%)	Non-health related n (%)		
Aware of NTDs			3.072	0.080
Yes	180(80.0)	45(20.0)		
No	5(5.9)	80(94.1)		
Knows the meaning of acronym NTD			3.565	0.059
Correct	103(62.4)	62(37.6)		
Incorrect	52(86.7)	8(13.3)		
Source of information on NTD			2.219	0.330
School/seminar	161(71.6)	32(28.4)		
News	7(38.9)	11(61.1)		
Social media	4(28.6)	10(71.4)		
Has seen anyone with NTD?			2.889	0.089
Yes	103(75.2)	34(24.8)		
No	75(85.2)	13(14.8)		
Are all NTDs public health problems?			8.802	0.030
Yes	102(72.3)	39(27.3)		
No	68(81.0)	16(19.0)		
Knows any NTD programme?			0.416	0.519
Yes	130(86.1)	21(13.9)		
No	48(64.9)	26(35.1)		
Name three NTD control programmes			13.464	0.004
Named one	14(50.0)	14(50.0)		
Named two	27(73.0)	10(27.0)		
Named three/more	29(100.0)	0(0.0)		
Incorrect names	48(84.2)	9(15.8)		
How many NTDs are there?			10.416	0.005
17	33(100.0)	0(0.0)		
20 (correct answer)	12(100.0)	0(0.0)		
I don't know	133(73.8)	47(26.2)		
How many NTDs are considered ‘tool ready’?			2.282	0.319
7 (correct answer)	14(77.8)	4(22.2)		
9	56(50.0)	56(50.0)		
I don't know	58(61.1)	37(38.9)		
Do you know any ‘tool ready’ NTD?			0.188	0.043
Yes	37(78.7)	10(21.3)		
No	134(75.3)	44(24.7)		
If yes to above, name five tool ready NTDs			2.442	0.486
Named at least one	27(100.0)	0(0.0)		
Named at least two	8(80.0)	2(20.0)		
Named at least five and above	8(80.0)	2(20.0)		
How many of the NTDs is/are vaccine preventable?				
1	12(85.7)	2(14.3)	3.548	0.001
3 (correct answer)	3(75.0)	1(25.0)		
I don't know	114(55.1)	93(44.9)		
Aware of the ‘overarching 2030 global targets’ concerning NTDs?				
Yes	16(61.5)	10(38.5)	0.074	0.786
No	132(66.3)	67(33.7)		

## Discussion

This study assessed awareness, knowledge, and perceptions regarding NTDs among personnel in a tertiary healthcare facility, while identifying critical gaps.

The largest age groups were 31–40 and 41–50 years (37.7% each), contrasting with younger age distributions reported in Ekiti (13) and Abuja (11). Nearly all respondents (97.4%) had tertiary education, reflecting the institutional setting. More than half (59.7%) were from health-related disciplines, consistent with other research among healthcare providers (10,12), medical and nursing students (14,15), and general populations (16,17).

Awareness levels were moderate: 72.6% had heard of NTDs, and 73.3% correctly identified the acronym. However, only 42.6% of those aware demonstrated good knowledge. Similar gaps have been reported in Abuja (11), Cameroon (16), and Egypt (15). The heavy reliance on schools and seminars (85.8%) as information sources, with limited input from mass media, indicates a narrow channel of knowledge dissemination. In contrast, studies in Egypt found social media to be a primary source (15). Expanding NTD education through media campaigns could enhance awareness among non-health professionals. Social media–based campaigns, such as those described by Aisiri et al. (17), demonstrate promise.

Although 60.9% had seen someone with an NTD and 62.7% recognized all NTDs as public health problems, deeper technical knowledge was limited. Only 5.3% knew the correct total number of NTDs, and just 8.0% could identify the number of tool-ready NTDs. Awareness of vaccine-preventable NTDs (1.8%) and global NTD targets (11.6%) was alarmingly low. These findings mirror gaps reported in Nigerian and regional studies (10,13,16,18).

Respondents generally recognized systemic preventive measures such as sanitation, poverty reduction, and healthcare funding, aligning with evidence from Mozambique (19) and Ethiopia (20) where poor knowledge of transmission and prevention hindered control efforts. However, endorsement of legislation (53.2%) was relatively low, possibly reflecting limited understanding of existing policy frameworks (22).

Health-related respondents consistently outperformed non-health-related respondents in awareness and knowledge, a finding echoed by studies across Asia where medical students demonstrated higher understanding of NTDs (14). This suggests discipline-specific exposure and underlines the need for cross-disciplinary workplace education, continuous professional development, and targeted public health campaigns

In conclusion, this study revealed general awareness of NTDs but significant gaps in technical knowledge crucial for prevention and control. These deficits undermine public health education, advocacy, and program implementation. Addressing them through targeted training, media-based education, and community-focused campaigns can strengthen NTD control efforts.

Healthcare personnel, both clinical and support staff are central to successful NTD programs. Strengthening their capacity in endemic regions will enhance patient outcomes, interrupt transmission cycles, and contribute to resilient healthcare systems capable of advancing toward global NTD elimination targets.

## Figures and Tables

**Table 3:** Responses to measures for preventing/controlling NTDs

Variable (n=310)	Yes, n(%)	No, n(%)
Equitable health to all (correct answer is yes)	213 (68.7)	97 (31.3)
Health education on NTDs (correct answer is yes)	233 (75.2)	77 (24.8)
Poverty reduction (correct answer is yes)	220 (71.0)	90 (29.0)
Legislation on NTD prevention (correct answer is yes)	165 (53.2)	145 (46.8)
Improved sanitation (correct answer is yes)	220 (71.0)	90 (29.0)
Funding of NTD agencies (correct answer is yes)	218 (70.3)	92 (29.7)
Building and equipping hospitals (correct answer is yes)	218 (70.3)	92 (29.7)
Change of government (correct answer is no)	50 (16.1)	260 (83.9)
Curtailing people's movement (correct answer is no)	75 (24.2)	(75.8)

**Figure 1:**
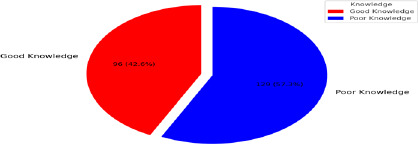
Knowledge of NTDs of the respondents
